# *c-fos* induction in the choroid plexus, tanycytes and pars tuberalis is an early indicator of spontaneous arousal from torpor in a deep hibernator

**DOI:** 10.1242/jeb.247224

**Published:** 2024-05-23

**Authors:** Fredrik A. F. Markussen, Fernando Cázarez-Márquez, Vebjørn J. Melum, David G. Hazlerigg, Shona H. Wood

**Affiliations:** Arctic Seasonal Timekeeping Initiative (ASTI), Arctic Chronobiology and Physiology, Department of Arctic and Marine Biology, BFE, UiT – The Arctic University of Norway, Tromsø, NO-9037, Norway

**Keywords:** Hibernation, Thermoregulation, *c-fos*, Golden hamster, Choroid plexus, Tanycytes, Dorsomedial hypothalamus, Pars tuberalis

## Abstract

Hibernation is an extreme state of seasonal energy conservation, reducing metabolic rate to as little as 1% of the active state. During the hibernation season, many species of hibernating mammals cycle repeatedly between the active (aroused) and hibernating (torpid) states (T–A cycling), using brown adipose tissue (BAT) to drive cyclical rewarming. The regulatory mechanisms controlling this process remain undefined but are presumed to involve thermoregulatory centres in the hypothalamus. Here, we used the golden hamster (*Mesocricetus auratus*), and high-resolution monitoring of BAT, core body temperature and ventilation rate, to sample at precisely defined phases of the T–A cycle. Using *c-fos* as a marker of cellular activity, we show that although the dorsomedial hypothalamus is active during torpor entry, neither it nor the pre-optic area shows any significant changes during the earliest stages of spontaneous arousal. Contrastingly, in three non-neuronal sites previously linked to control of metabolic physiology over seasonal and daily time scales – the choroid plexus, pars tuberalis and third ventricle tanycytes – peak *c-fos* expression is seen at arousal initiation. We suggest that through their sensitivity to factors in the blood or cerebrospinal fluid, these sites may mediate metabolic feedback-based initiation of the spontaneous arousal process.

## Teaser

**Highlighted Article:** Three non-neuronal sites previously linked to control of metabolic physiology over seasonal and daily time scales show a peak in *c-fos* expression at the initiation of arousal from torpor; these sites may mediate metabolic feedback-based initiation of the spontaneous arousal process.

## INTRODUCTION

Hibernation is a seasonally regulated process of energy conservation achieved through extreme shut down of metabolic energy expenditure, sometimes to as little as 1% of that in the euthermic state, allowing species to survive extended winter periods of low energy availability (Kenagy et al., 1989; McKee and Andrews, 1992; Ruf and Geiser, 2015). Hibernation is characterised by multi-day bouts of torpor during which metabolic rate is suppressed and core body temperature (*T*_b_) falls, in some species to within a few degrees of ambient temperature (*T*_a_), while breathing and heart rate slow to an extent not expected to support life in endothermic organisms (Lyman et al., 1982).

In many hibernating species, torpor bouts are separated by relatively short arousal episodes during which *T*_b_ rises back to euthermic levels, so that the hibernation season as a whole comprises a series of torpor–arousal (T–A) cycles. This presents a paradox: why would an organism undergo a costly arousal if the ultimate goal of hibernation is to save energy? As homeostatic processes consume energy to maintain healthy cellular environments, one scenario is that arousal episodes allow restoration of healthy cellular conditions by reactivating aspects of homeostasis that are compromised or suspended during torpor.

While this narrative provides plausible ultimate reasoning for the evolution of periodic arousal, it does not address the proximate mechanisms by which T–A cycling is controlled. Here, two basic models can be considered: a clock-based mechanism and a metabolic feedback-based mechanism. In its most explicit formulation, the former is conceived as an extension of circadian function (Körtner and Geiser, 2000; Malan, 2010). The hypothalamic circadian pacemaker in the suprachiasmatic nucleus (SCN) controls daily *T*_b_ cycles in euthermic mammals and the expression of daily torpor episodes in Siberian hamsters (Körtner and Geiser, 2000; Ruby and Zucker, 1992; Ruf et al., 1989). A role for circadian control mechanisms in multiday T–A cycling is not favoured, however. Except for the first torpor bout of the season, T–A cycles show no circadian organisation (Hut et al., 2002; Williams et al., 2012, 2017), and SCN molecular clock gene oscillations flatten out (Ikeno et al., 2017; Revel et al., 2007). Malan proposed a poorly temperature compensated circadian mechanism in which slow running of the circadian oscillation at low temperatures determines timing of arousals (Malan, 2010; Malan et al., 2018), but this conjecture fails to account for changes in T–A cycle periodicity, particularly at the beginning and end of the hibernation season, or for the finding that the period of T–A cycling is the same in wild-type Syrian hamsters and in *tau* mutant hamsters in which the circadian clock runs fast (Oklejewicz et al., 2001). Hence, current models focus on the second possibility, namely that T–A cycle regulation stems from accumulation (or depletion) during the torpid state of metabolite(s) which act as signals to stimulate the arousal process, and that levels of these metabolic signals are then reset during arousal, permitting torpor re-entry (reviewed in van Breukelen and Martin, 2015). As arousal is initiated by combined increases in pulmonary-cardio-vascular activity and brown adipose tissue (BAT) activation, this framing places an emphasis on metabolic signals that access brain mechanisms controlling sympathetic stimulation of these tissues.

The circuits controlling BAT thermogenic activity in euthermic rodents include the temperature-sensitive pre-optic area, which acts via the dorsomedial hypothalamus (Cannon and Nedergaard, 2004; Morrison, 2016). In mice, chemo/opto-genetic manipulation of the activity of neurons within the pre-optic area can suppress *T*_b_ and metabolic rate (Hrvatin et al., 2020; Takahashi et al., 2020) to produce a torpor-like state, suggesting that metabolic feedback to these sites could be involved in natural torpor regulation. Therefore, a link between peripheral communication to the brain via factors in the circulation and cerebrospinal fluid (CSF) is an attractive option for the acute regulation of a T–A cycle. A study in the Siberian chipmunk (*Eutamias sibiricus*) identified a ‘hibernation factor’, produced in the liver and transported to the brain via the choroid plexus; however, changes during a T–A cycle were not investigated, and only seasonal changes were reported (Kondo et al., 2006). Furthermore, these seasonal changes are also reported in the non-hibernating cow (*Bos taurus*); therefore, a direct link to hibernation is unlikely (Seldin et al., 2014).

To identify key signals controlling spontaneous T–A cycling, it is necessary to overcome the challenge of repeatable sampling immediately prior to and in the earliest stages of spontaneous arousal from torpor. Because this is difficult, ‘forced’ arousal by handling or by changing room temperature has been employed (for example: Regan et al., 2019). Unfortunately, this necessarily alters the dynamics of the T–A cycle and causes stress (Tähti and Antti, 1977; Utz and van Breukelen, 2013), potentially confounding attempts to characterise brain mechanisms involved in spontaneous T–A cycling. Bratincsák et al. (2007) combined *T*_b_ measurement and expression of the immediate early gene *c-fos* to explore brain changes during spontaneous T–A cycles in thirteen-lined ground squirrels. While this revealed *c-fos* changes in many brain sites, the use of *T*_b_ as the sole indicator of arousal status inevitably misses the early stages of the arousal process, which may precede core *T*_b_ by almost 1 h (Markussen et al., 2021).

Here, we have developed an enhanced telemetric approach based on BAT temperature combined with *T*_b_ and ventilation rate to characterise T–A cycling at high temporal and temperature resolution in hibernating golden hamsters, a species showing highly reproducible T–A cycles (Chayama et al., 2016; Lyman, 1948; Lyman et al., 1982). Using *c-fos* RNA expression, we sought to capture the early dynamics of brain activation during spontaneous T–A cycling. Our data reveal changes in the choroid plexus, pars tuberalis and tanycytes lining the 3rd ventricle of the hypothalamus, as the earliest brain indicators of initiation of spontaneous arousal.

## MATERIALS AND METHODS

### Animals and experimental set up

All experimental procedures were carried out at the animal facility of Arctic Chronobiology and Physiology at UiT – The Arctic University of Norway. All procedures and experiments were approved under national legislation by the Norwegian Food Safety Authority under the laboratory animal administration's supervision and application system ID: 24904. Golden hamsters can be induced to hibernate in the laboratory when given the correct photoperiodic history and permissive temperatures. In climate- and light-controlled rooms, 5 month old male golden hamsters (*n*=30, *Mesocricetus auratus*, strain RJHan:AURA; Janvier Labs, Le Genest-Saint-Isle, France) raised on a long photoperiod since birth (14 h light, 10 h dark), were kept in sibling groups with *ad libitum* access to food and water, and housed under long photoperiod (LP: 16 h light:8 h dark) and 21°C for 4 weeks. Prior to induction of hibernation, Anipill^®^ temperature loggers (Animals-monitoring, Hérouville Saint-Clair, France) and IPTT-300 temperature transponder tags (BMDS/Plexx BV, Elst, The Netherlands) were implanted under surgical anaesthesia in the abdominal cavity and interscapular brown fat deposit, respectively. The animals were then single housed and allowed 2 weeks to recover under LP. To induce hibernation, single-housed hamsters were switched to short photoperiod (SP: 8 h light:16 h dark) for 4 weeks at 21°C (SP-warm) before lowering the ambient temperature to ∼7°C (SP-cold, mean±s.d. 7.4±0.53°C).

### Physiological and behavioural monitoring

To reduce stress and disturbance to the animals during the SP-cold phase, a select group of trained personnel conducted daily monitoring, thereby maintaining a stable and controlled environment conducive to animal welfare.

Real-time monitoring of interscapular BAT temperature (*T*_iBAT_) was done using a mix of proprietary hardware and readily available electronics components using open-source practices, together with an open-source software stack. Iterative reads of *T*_iBAT_ were done using a DAS-8027 reader (BMDS/Plexx BV), which was fixed in position above the scapular region of the hibernating hamster. The reader was configured to operate in automatic scanning mode, recording from the IPTT-300 tag every 3–5 s. The sensitivity of the IPTT-300 was 0.1°C with an accuracy of 0.5°C when corrected by previously described standardised in-house calibration tests (Markussen et al., 2021). Data acquisition from the reader was wirelessly transmitted though the BMDS communications module to a computer in a separate room. The data were written into a text document using Atom text editor (https://github.com/atom/atom) and changes were automatically saved. A program written using NodeRED v.19.9.0 (OpenJS Foundation) monitors this file and automatically extracts the last row of information as soon as data are written, formats it, and pushes the data into an InfluxDB v.1.8.1 (InfluxData Inc., San Francisco, CA, USA) database housed in the local computer (JavaScript for replication of the NodeRED program is available from https://github.com/ShonaWood/cFOSGHam). *T*_a_ was also continuously monitored using RuuviTag Bluetooth sensors (Ruuvi Innovations Ltd, Riihimäki, Finland), and data were pushed to Influx DB using NodeRed. InfluxDB data were accessed and displayed using Grafana v.10.0.1 (GrafanaLabs, New York, NY, USA); this allowed real-time monitoring from any computer or smart phone. For each individual torpor bout, we established a torpor baseline for *T*_iBAT_ temperature by performing live continuous calculations in the Grafana interface for at least 5 h, some 20 h before the predicted arousal time for the animal (based on average bout duration; Fig. S1D). To establish a torpor baseline, we subtracted *T*_a_ from *T*_iBAT_ to give a thermal distance (TD), calculated every 3–5 s for at least 5 h. A mean TD was then calculated and used to ensure each torpor bout had a tailored torpor baseline of zero, which accounts for any changes in *T*_a_. Across all torpor bouts, the TD was a mean±s.d. of 0.71±0.19°C. When monitoring arousal, we calculated the change from torpor baseline (Δ*T*); mathematically, this is simply expressed as:
(1)

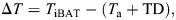
where TD is the mean distance between *T*_a_ and *T*_iBAT_ during torpor, established for each individual and each torpor bout prior to arousal. Departures from Δ*T* were then used to detect animals spontaneously arousing in addition to the fulfilment of the criteria of arousal stated below.

In parallel, an infrared-sensitive camera (Raspberry Pi Camera Module 2 NoIR, Raspberry Pi Foundation, Cambridge, UK) connected via Raspberry Pi (model 3B+, Raspberry Pi Foundation) was used to stream a video feed to monitor and record animal activity and ventilation frequency using software developed by Singh et al. (2019).

We then analysed 12 T–A cycles from 6 individuals to select physiological sampling criteria, with the goal of reducing inter-individual variation and identifying the earliest possible reliable predictors of arousal. Our criteria were: animals entering torpor (ENT<25°C) must demonstrate a minimum of a 12 h inter-bout euthermic (IBE) duration, followed by a steady decrease in observed Anipill recorded *T*_b_ to below 25°C. Additionally the IPTT read must be below 25°C and the animal must be in curled up torpid posture. Torpid animals (T-40 h) were sampled 40 h after ENT<25°C, if *T*_b_ and *T*_iBAT_ were within 2°C of *T*_a_ and ventilation frequency was less than 2 breaths min^−1^. Arousal 1 (AΔ0.5°C) was defined only if an animal had entered torpor and remained torpid for longer than 40 h, and subsequently showed a ventilation frequency >10 breaths min^−1^ and a 0.5°C change in *T*_iBAT_ from torpor baseline (Δ*T*). Arousal 2 (AΔ3°C) followed the same criteria as arousal 1 but was sampled at Δ*T*=3°C. An animal was defined as being IBE if it had entered torpor, remained in torpor for longer than 40 h, subsequently aroused and then reached a *T*_b_ of 30°C. Animals were sampled 1 h (IBE-1 h) and 12 h (IBE-12 h) after euthermia was reached.

Prior to sampling, all animals had shown at least 3 full T–A cycles; these data were used to verify the sampling criteria prior to sampling in the 4th T–A cycle. On reaching the above criteria, the animals were euthanised by cervical dislocation. The brain was rapidly dissected and frozen in isopentane before storage at −80°C until sectioning.

### *In situ* hybridisation

Regions of interest (ROI), the pre-optic area (Bregma ca. 0.8 to 0.95) and mediobasal hypothalamus (Bregma ca. −2.5 to −2.8), were collected in 14 µm sections onto Fisherbrand Superfrost Plus slides using a Leica CM3050S cryostat and stored at −80°C until further processing.

On the day of hybridisation, sections were dried for 15 min at 50°C before fixation with 4% formaldehyde solution for 20 min. Sections were acetylated and delipidated using 1% (v/v) triethylamine (Sigma, 90335) with 0.25% (v/v) acetic anhydride (Sigma, 539996) and 0.1% (v/v) Triton X-100 (Sigma, X100) in nuclease-free water, respectively. The sections were hybridised with a 200 ng ml^−1^ *c-fos* antisense probe (a gift from Valerie Simonneaux and Paul Klosen, University of Strasbourg, 793-1193 of GenBank accession no. XM_005086369.4) overnight at 58°C in hybridisation buffer containing 5× Denhardt's solution (Sigma, D2532), 0.25 mg ml^−1^ yeast tRNA (Roche, 10109525001), 0.2 mg ml^−1^ fish sperm ssDNA (Roche, 11467140001), 5× saline-sodium citrate (SSC, Sigma, 8310-OP) and 50% (v/v) formamide (Sigma, F9037) in nuclease-free water (Sigma, F9037). Following hybridisation, washes were made in decreasing concentrations of SSC (5× and 2×) at 55°C, 50% formamide in 0.2× SSC at 55°C, and 0.2× SSC at room temperature. After blocking using 1% Roche blocking solution (Roche, 11096176001), sections were incubated for 3 h with Anti-Digoxigenin-AP Fab fragments (dilution 1:3000; Roche, 11093274910) at room temperature, followed by washes in A-dig and alkaline phosphate buffer. The signal was then visualised by incubating the slides for 48 h in a solution containing 0.33 mg ml^−1^ NBT (Nitro-blue tetrazolium chloride, Roche, 11383213001) and 0.165 mg ml^−1^ BCIP (5-bromo-4-chloro-3-indolyl phosphate, Roche, 11383221001) in alkaline phosphatase buffer. Once the signal had developed properly, the slides were prepared for immunohistochemical processing to visualise vimentin and cell nuclei. After three 5 min washes in PBS pH 7.6 containing 0.05% (v/v) Tween-20 (PBST, Sigma, P1379), the slides were incubated in blocking buffer containing PBST, 0.1% (w/v) cold water fish skin gelatin (Sigma, G7041), 0.1% (w/v) bovine serum albumin (Sigma, A2153) and 3 mmol l^−1^ glycine (Sigma, G7126) for 1 h at room temperature, followed by overnight incubation at 4°C in blocking buffer containing mouse anti-vimentin antibody (Sigma, MAB3400) diluted 1:2000. After four 10 min washes in PBST at room temperature, the secondary antibody was applied by incubating the slides for 1 h at room temperature with blocking buffer containing Alexa Fluor 647-conjugated goat anti-mouse antibody (1:1000, Sigma, SAB4600354). The slides were then briefly rinsed in PBST before a 10 min incubation in PBST containing 1:2000 SYTOX Orange (Thermo Fisher, S11368), followed by four 10 min washes in PBST and a final rinse in PBS. The slides were then coverslipped and sealed using nail polish and 1.5H coverslips over antifade solution containing 90% (v/v) glycerol (Sigma, G7757), 10% (v/v) PBS and 2.5% (w/v) diazabicyclo 2.2.2 octane (DABCO, Sigma, 290734).

The slides were scanned using an Olympus VS120 slide scanning microscope at the core microscopy facility at UiT. Overview images of the sections were obtained using a Cy3 filter set, then ROI were marked up in the OlyVIA software and, by using the ×40 objective, high-resolution images were obtained. The images were analysed using QuPath v.0.4.3 by annotating ROI. The number of cells within each ROI was detected and quantified using the SytoxO channel in the standard cell detection plugin in QuPath. To quantify the amount of *c-fos* within each ROI, background intensity in a 500 µm^2^ area on each section was used to calculate a threshold for detection using the signal to background ratio. Signal above threshold was determined to be *c-fos* positive. To be classified as a *c-fos*-positive cell, an above-threshold signal within cell boundaries was required. The percentage of *c-fos-*positive cells within each ROI was calculated. Figures were made by exporting ROI to ImageJ v.1-54f, where they were assembled using the FigureJ package.

### Statistical analysis

The physiological and cell counts data were analysed in R v.4.3.1. The scripts for the analysis and the data are available from GitHub (https://github.com/ShonaWood/cFOSGHam).

Time of day preference was assessed using Rayleigh test of circular uniformity using R. Significant differences between numbers of *c-fos*-positive cells was assessed by one-way AVOVA and *post hoc* testing by Dunnett's multiple comparisons test using R.

## RESULTS

### Reproducible induction of hibernation and reliable prediction of torpor entry and arousal

**Fig. 1.**
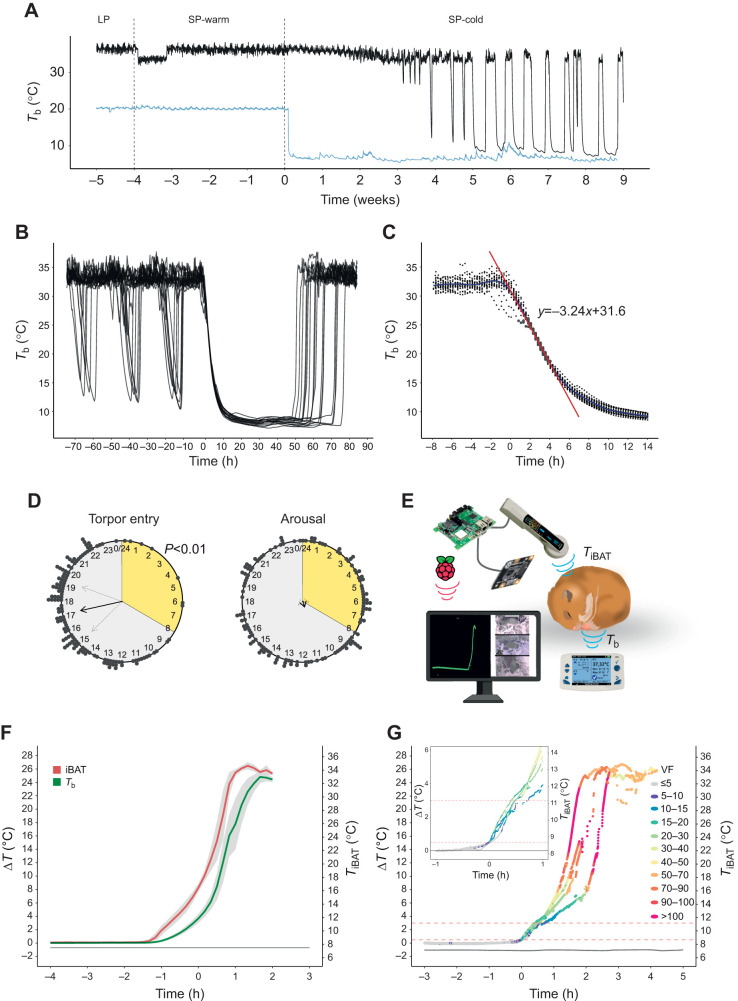
**Physiological monitoring of the golden hamster during hibernation.** (A) Representative core body temperature trace (*T*_b_, black) and ambient temperature (*T*_a_, blue; data collected every second and binned into 10 min averages) over the whole experiment. Hamsters were kept on a long photoperiod (LP, 16 h light:8 h dark; *T*_a_=21°C) for 6 weeks, then moved to a short photoperiod (SP, 8 h light:16 h dark) and warm conditions (SP-warm, *T*_a_=21°C) for 4 weeks. Finally, the *T*_a_ was lowered (SP-cold), achieving a mean±s.d. of 7.4±0.53°C for the entire SP-cold period. At week 6, a heatwave resulted in an average increase of 1.6°C for 5 days. The vertical dotted lines indicate when the changes in light or temperature conditions occurred. Note the drop in *T*_b_ in the first week of SP; this was due to the Anipill logger shifting into the testis sack, which resolved itself. (B) *T*_b_ traces of 18 hamsters synchronised to first torpor bout entry, illustrating test-drops prior to torpor–arousal (T–A) cycling and the first T–A cycle. (C) *T*_b_ of entry into torpor from 18 individuals to explore variation in entry dynamics. The red line is a regression analysis for the steepest part of the slope, showing an average maximal cooling rate of −3.24°C h^−1^. (D) Raleigh plots showing the time of entry into torpor and arousal from 28 individuals. Photoperiod is represented by grey for dark and yellow for light. The black dots are an individual arousal or entry event. The black arrow indicates the mean time of entry or arousal, and the light grey arrows indicate the s.d. The length of the arrow represents mean resultant length as an indicator of concentration around the mean, therefore reflecting the statistical significance. The Raleigh statistical test was used to test for a time-of-day preference in arousal and torpor entry. Only torpor entry was significant (*P*<0.01). (E) A schematic diagram of the remote monitoring system for arousal from torpor. IPTT-300 tags were used to monitor interscapular brown adipose tissue temperature (*T*_iBAT_) dynamics. A live video feed using a Raspberry Pi and camera module measured ventilation frequency. Anipill loggers were used to monitor *T*_b_. *T*_iBAT_ was continuously read every 3–5 s and all data were parsed to a networked database immediately accessible to local networked devices. (F) The change in *T*_iBAT_ (red) and *T*_b_ (green) from torpor baseline *T*_b_ (Δ*T*, left axis) and the actual *T*_iBAT_ (right axis) from 10 representative arousals. Mean values are shown with s.d. in grey shading. (G) A closer analysis of *T*_iBAT_ dynamics during arousal for four representative arousals. The colour of the dots reflects ventilation frequency (VF, in breaths min^−1^). The change in *T*_iBAT_ from torpor baseline (Δ*T*) is plotted on the left axis and the actual *T*_iBAT_ is plotted on the right axis. The dashed red lines indicate 0.5°C and 3°C change from torpor baseline. Occasionally, the scanner lost contact with the tag, as a result of shivering (approximately 16°C from torpor baseline), resulting in missing data points. Inset: the first hour of arousal on an expanded scale.

We switched animals from a LP (16 h light:8 h dark) at 21°C to a SP (8 h light:16 h dark) at 21°C (SP-warm) for 4 weeks, then we lowered the temperature to ∼7°C (SP-cold) and monitored *T*_b_ continuously (Fig. 1A). Within 8 weeks of exposure to SP-cold conditions, 80% of our animals had commenced multi-day T–A cycling; this increased to 93% by 12 weeks (Fig. S1A). Similar to previous studies, we observed a gradual resetting of mean (±s.d.) *T*_b_ from 36.4±0.83°C to 33.4±0.98°C after 4 weeks in SP-cold, indicating a seasonal preparatory phase prior to the expression of hibernation (Chayama et al., 2016) (Fig. 1A; Fig. S1B). We also observed so-called ‘test-drops’ (Fig. 1A,B) (Sheriff et al., 2012) in 86% of our animals prior to the initiation of T–A cycling. During test drops, the mean *T*_b_ was 21.4±7.35°C and the mean test-drop duration was only 5.3 h. Both entry into and arousal from test drops showed circadian organisation (Fig. S1C). Not all animals showed test-drops prior to multiday T–A cycling, indicating that they are not a pre-requisite for hibernation.

Once initiated, T–A cycles were reproducible within and between animals, with *T*_b_ falling to within an average of 0.75°C of *T*_a_. Torpor bout length increased from 56.5±9.9 h at the first bout to a maximum of 75.5±8.9 h by the sixth bout, while the IBE interval decreased from 61.2±38.3 h to 22.9±7.86 h (Fig. S1D).

The *T*_b_ dynamics of the entry phase to torpor followed an inverse sigmoidal trajectory (Fig. 1C). This was somewhat variable between 33 and 26°C, with some animals showing a ‘step-wise’ entry pattern; thereafter, *T*_b_ declined approximately linearly at a maximum cooling rate of −3.24°C h^−1^, until about 15°C, when the approach to nadir values slowed (Fig. 1C). Initiation of torpor was more likely to occur in the dark phase (approximately 17–22 h after lights on; *P*<0.01 by Rayleigh test of circular uniformity) (Fig. 1D).

Consistent with previous studies, arousal from torpor showed no circadian organisation (Fig. 1D) (Hut et al., 2002; Oklejewicz et al., 2001; Williams et al., 2012, 2017). As *T*_b_ is not a good early indicator of arousal (Markussen et al., 2021), we used IPTT-300 tags to measure *T*_iBAT_ every 3–5 s, and monitored ventilation frequency by video-streaming (Fig. 1E). During spontaneous arousal, increases in *T*_iBAT_ preceded increases in *T*_b_ by up to 40 min (Fig. 1F). We noted inter-animal variability in the time to rewarm from 4°C above torpor baseline (Δ*T*) to 10°C above (range 30–65 min). Once above 10°C from torpor baseline (Δ*T*) rewarming to euthermy followed a similar trajectory and time line across individuals, with ventilation rates rapidly increasing towards peak values, together with a maximum rewarming speed of *T*_iBAT_ and *T*_b_ (+18.3°C h^−1^). Despite variability in its initiation, arousal was completed within 2.5–3 h in all animals (Fig. 1G). To determine the earliest possible time at which we could reliably predict an arousal event and clearly differentiate it from animals thermoregulating during torpor, we monitored 12 separate arousals. We found that an increase in *T*_iBAT_ of 0.5°C above torpor baseline coupled with a ventilation frequency >10 breaths min^−1^ was the earliest reliable predictor of a forthcoming arousal event, approximately 40 min before a detectable change in *T*_b_ (Fig. 1F,G).

### The dorsomedial hypothalamus expresses *c-fos* during entry into torpor

**Fig. 2.**
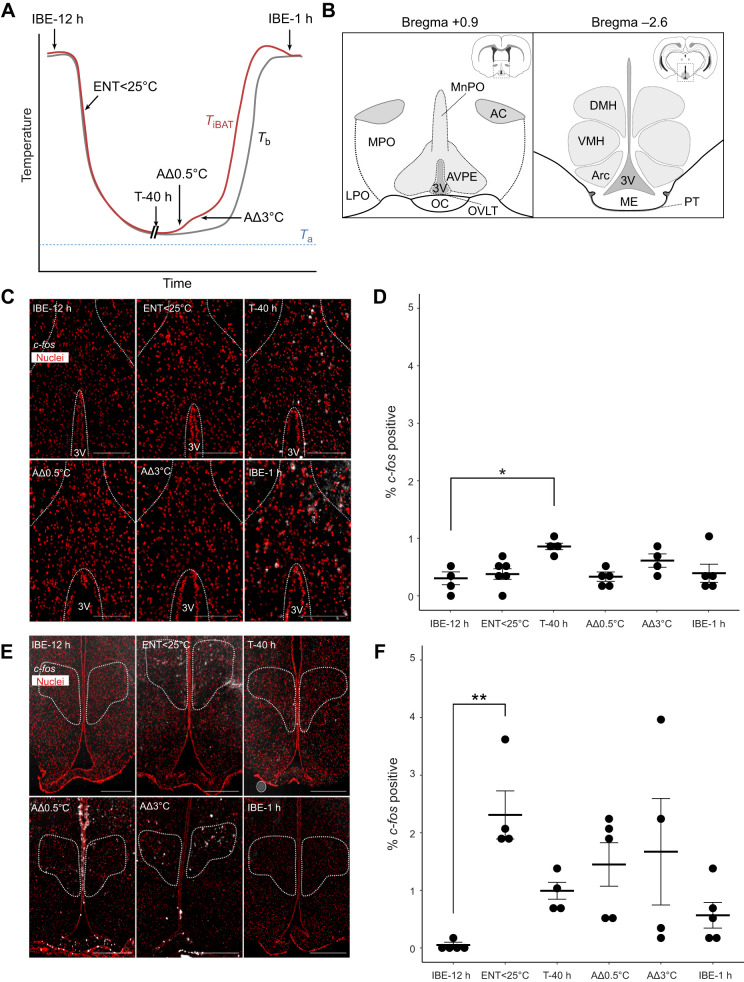
***c-fos* expression in the pre-optic area and dorsomedial hypothalamus during T–A cycling.** (A) A schematic drawing showing *T*_iBAT_ (red) and *T*_b_ (grey) and the sampling points taken during the T–A cycle. Interbout euthermic animals were sampled either 1 or 12 h after arousal (IBE-1 h, IBE-12 h). Animals entering torpor were sampled when *T*_b_ and *T*_iBAT_ were less than 25°C (ENT<25°C). Torpid animals were sampled 40 h into the torpor bout (T-40 h). Early arousal animals were defined by deviation in *T*_iBAT_ from the torpor baseline *T*_b_ of either 0.5°C or 3°C (AΔ0.5°C and AΔ3°C). (B) Brain regions of interest defined by the golden hamster brain atlas. Left panel: Bregma +0.9 in the pre-optic hypothalamic area (POA): MnPO, median preoptic area; AVPE, anteroventral periventricular area; MPO, medial preoptic area; LPO, lateral preoptic area; OVLT, organum vasculosum lamina terminalis; 3V, third ventricle; OC, optic chiasm; AC, anterior commissure. Right: Bregma −2.6 in the mediobasal hypothalamus: DMH, dorsomedial hypothalamus; VMH, ventromedial hypothalamus; Arc, arcuate nucleus; 3V, third ventricle; ME, median eminence; PT, pars tuberalis. (C) Representative images of *in situ* hybridisation for *c-fos* (white), with SYTOX Orange staining to show nuclei (red), in the pre-optic area, indicated by a white outline, for each group defined in A. Scale bars: 500 µm. (D) Quantification of the percentage of *c-fos*-positive cells in the pre-optic area. Each circle represents one animal, with three sections per animal quantified. Error bars represent the s.e.m. Results of one-way ANOVA and *post hoc* testing by Dunnett’s multiple comparisons test are shown; *P≤0.05. (E) Representative images of *in situ* hybridisation for *c-fos* (white), with SYTOX Orange staining to show nuclei (red), in the dorsomedial hypothalamus, indicated by a white outline, for each group defined in A. Scale bars: 500 µm. (F) Quantification of the percentage of *c-fos*-positive cells in the dorsomedial hypothalamus. Each circle represents one animal, with three sections per animal quantified. Error bars represent the s.e.m. Results of one-way ANOVA and *post hoc* testing by Dunnett’s multiple comparisons test are shown; **P≤0.005.

We measured RNA expression of the immediate early gene *c-fos* by *in situ* hybridisation and validated our probes using a light pulse paradigm and staining of the suprachiasmatic nucleus (Fig. S2A) (Earnest et al., 1990). Then, we sampled animals that had undergone at least three full T–A cycles at the following phases (for details of calculation, see Materials and Methods): torpor entry (ENT>25°C), 40 h torpid (T-40 h), the earliest spontaneous arousal (AΔ0.5°C), early spontaneous arousal prior to the phase of high inter-animal variation (AΔ3°C), and interbout euthermic animals after 1 h (IBE-1 h) and 12 h (IBE-12 h) (Fig. 2A).

**Fig. 3.**
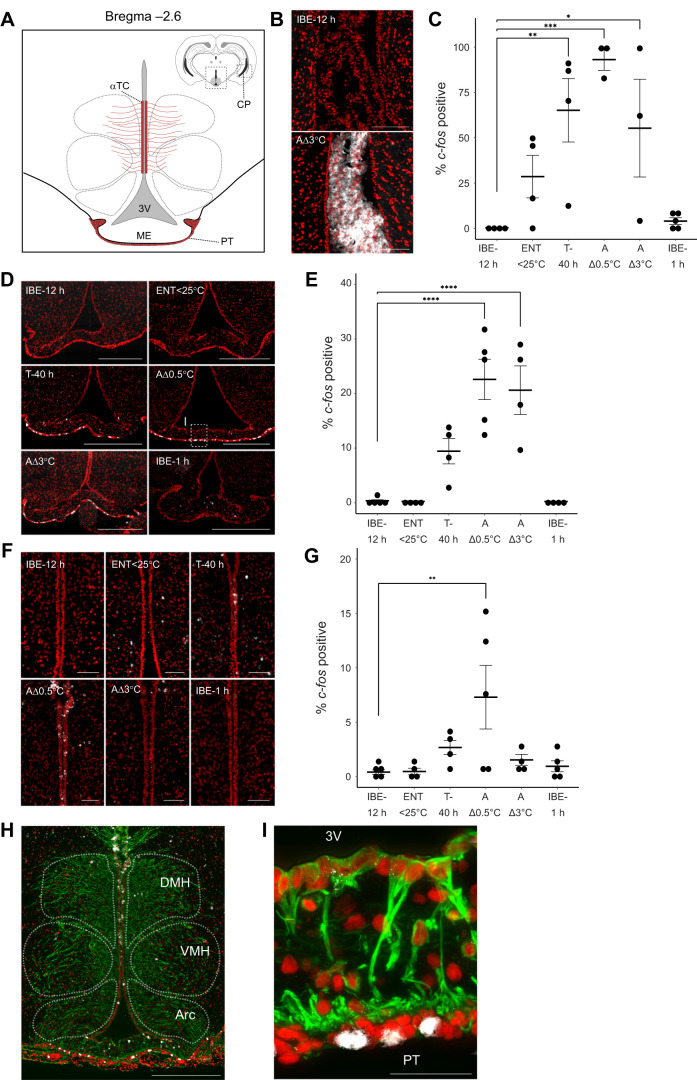
***c-fos* expression in the choroid plexus, pars tuberalis and tanycytes during early arousal from torpor.** (A) A schematic drawing indicating Bregma −2.6 and the location of the choroid plexus (CP), pars tuberalis (PT) and α-tanycytes (αTC). ME, median eminence; 3V, third ventricle. Grey dotted lines indicate the regions of the hypothalamic nuclei. Note, the choroid plexus is in the lateral ventricles and is therefore displayed in the inset relative to the hypothalamic region shown. (B) Representative images of *in situ* hybridisation for *c-fos* (white), with SYTOX Orange staining to show nuclei (red), in the choroid plexus, for IBE-12 h and AΔ3°C. Scale bars: 500 µm. (C) Quantification of the percentage of *c-fos*-positive cells in the choroid plexus. Each circle represents one animal, with three sections per animal quantified. Error bars represent the s.e.m. Results of one-way ANOVA and *post hoc* testing by Dunnett’s multiple comparisons test are shown; *P≤0.05, **P≤0.005, ***P≤0.0005. (D) Representative images of *in situ* hybridisation for *c-fos* (white), with SYTOX Orange staining to show nuclei (red), in the pars tuberalis, for each group defined in Fig. 2A. The dotted box in the representative image for AΔ0.5°C indicates the region shown in I. Scale bars: 500 µm. (E) Quantification of the percentage of *c-fos*-positive cells in the pars tuberalis. Each circle represents one animal, with three sections per animal quantified. Error bars represent the s.e.m. Results of one-way ANOVA and *post hoc* testing by Dunnett’s multiple comparisons test are shown; ****P<0.0001. (F) Representative images of *in situ* hybridisation for *c-fos* (white), with SYTOX Orange staining to show nuclei (red), in the α-tanycyte region, for each group defined in Fig. 2A. Scale bars: 200 µm. (G) Quantification of the percentage of *c-fos*-positive cells in the α-tanycyte region. Each circle represents one animal, with three sections per animal quantified. Error bars represent the s.e.m. Results of one-way ANOVA and *post hoc* testing by Dunnett’s multiple comparisons test are shown; **P≤0.005. (H) Vimentin staining (green) for tanycyte processes in the mediobasal hypothalamus, *in situ* hybridisation for *c-fos* (white) and SYTOX Orange staining to show nuclei (red). The hypothalamic nuclei are indicated by white dotted lines. DMH, dorsomedial hypothalamus; VMH, ventromedial hypothalamus; Arc, arcuate nucleus. Scale bar: 500 µm. (I) A magnified image of the pars tuberalis shown in D during early arousal (AΔ0.5°C). Vimentin staining (green) for tanycyte processes, *in situ* hybridisation for *c-fos* (white) and SYTOX Orange staining to show nuclei (red). 3V, third ventricle. Scale bar: 50 µm.

Surveying the thermoregulatory circuits and comparing *c-fos* expression over these time points revealed that the pre-optic area (Fig. 2B) showed an approximately 0.5% increase the proportion of *c-fos*-positive cells after 40 h of torpor (T-40 h) compared with IBE-12 h (Fig. 2C,D; *P*<0.01, one-way ANOVA, Dunnett's multiple comparison test). Contrastingly, we saw significant changes in the number of *c-fos*-positive cells in the dorsomedial hypothalamus (Fig. 3B; DMH) during the entry phase of the T–A cycle compared with interbout euthermia (2.25% *c-fos*-positive cells in ENT>25°C versus to 0.1% in IBE-12 h; Fig. 2E,F; *P*<0.005, one-way ANOVA, Dunnett’s multiple comparison test). In the dorsomedial hypothalamus during arousal, *c-fos*-positive cells were detected but these changes were variable and therefore not significant. We also surveyed other neuronal centres in the hypothalamus [Fig. 2B; arcuate nucleus (Arc) and ventromedial hypothalamus (VMH)]; less than 1% of cells showed *c-fos* expression during torpor and almost no cells were *c-fos*-positive in the transition phases of the T–A cycle (Fig. S2B,C).

### *c-fos* induction in the choroid plexus, tanycytes and pars tuberalis, and during early arousal

We noted high expression of *c-fos* in the choroid plexus (Fig. 3A–C), with an average of 65% of choroid plexus cells showing positive staining for *c-fos* in torpid animals (T-40 h), rising to 93% in the earliest arousing animals (AΔ0.5°C), compared with 0.3% in IBE-12 h (Fig. 3C; IBE-12 h versus AΔ0.5°C, *P*=0.0005; IBE-12 h versus T-40 h, *P*=0.0063; one-way ANOVA, Dunnett’s multiple comparisons test). The circumventricular organs – the vascular organ of the lamina terminalis and the median eminence – showed approximately 5% of cells as *c-fos* positive during torpor and arousal but there was high inter-individual variation within the groups (Fig. S3A–D). Whilst surveying the median eminence, we noted that the pars tuberalis of the adjacent pituitary stalk (Fig. 3A) showed very high *c-fos* expression (22.6% of cells) at AΔ0.5°C (Fig. 3D,E; IBE12 h versus AΔ0.5°C, *P*<0.0001, one-way ANOVA, Dunnett’s multiple comparisons test). Similarly, α-tanycytes lining the 3rd ventricle of the mediobasal hypothalamus (Fig. 3A) showed increased *c-fos* expression in AΔ0.5°C (7.3%; Fig. 3F,G; IBE12 h versus AΔ0.5°C, *P*=0.0064, one-way ANOVA, Dunnett's multiple comparisons test). β-Tanycytes lining the 3rd ventricle but in closer proximity to the median eminence also showed an increase in *c-fos*-positive cells but to a lesser degree (Fig. S2E,F). Tanycytes have long projections into the hypothalamus and down to the pars tuberalis (Guerra et al., 2010; Güldner and Wolff, 1973; Rodríguez et al., 2005); we used vimentin to stain these projections, showing infiltration into the dorsomedial hypothalamus, VMH, Arc and pars tuberalis (Fig. 3H,I).

## DISCUSSION

We monitored spontaneous T–A cycles in the golden hamster at high temporal and temperature resolution to capture the initiation of spontaneous arousal from torpor. In two hypothalamic centres linked to thermoregulation, the pre-optic area and the dorsomedial hypothalamus (Cannon and Nedergaard, 2004; Morrison, 2016), no significant change in *c-fos* expression was seen during spontaneous arousal, and the only significant change observed was increased expression during torpor entry in the dorsomedial hypothalamus, potentially reflecting the tightly controlled cooling process (Florant and Heller, 1977). This contrasts with the pattern seen in three non-neuronal tissues, the choroid plexus, pars tuberalis and tanycytes, in which we detected the highest level of *c-fos* RNA expression when BAT temperature had risen only 0.5°C above torpor baseline. This indicates that these regions are sensitive to physiological changes at an early stage in the arousal process. This extends similar findings in the thirteen-lined ground squirrel (Bratincsák et al., 2007) and is consistent with the involvement of these sites in metabolic feedback regulation of T–A cycling.

The expression of *c-fos* in non-neuronal cells in the choroid plexus, the mediobasal hypothalamus and the pars tuberalis may reflect ionic changes that are not so clearly linked to ‘activation’ as is the case for neuronal *c-fos* expression (Lara Aparicio et al., 2022). Receptor stimulation leading to increases in intracellular calcium increase *c-fos* expression and potentially alter signalling to connected neuronal populations (Bolborea et al., 2020; Cruz-Mendoza et al., 2022; Dali et al., 2023; Guerra-Gomes et al., 2018; Lara Aparicio et al., 2022). Changes in hydro-osmotic balance can induce *c-fos* expression in the choroid plexus and other circumventricular organs (Menendez-Vallina et al., 2001). It is, however, possible that a translational block during torpor could increase *c-fos* expression through the removal of transcriptional negative feedback, but the highly time- and tissue-restricted nature of this response argues against this explanation and focuses attention on potential stimulation of non-neuronal cells contacting the CSF–blood–brain barrier through changes in CSF/blood composition during torpor.

The primary function of the choroid plexus is to produce and control the composition of CSF, maintaining a blood–CSF barrier that gates the exchange of metabolites and factors into the brain (Lun et al., 2015). The choroid plexus synthesises major transport proteins, notably the thyroid hormone carrier protein transthyretin (Duarte et al., 2020; Fame et al., 2023). This framing of choroid plexus function has clear parallels with the function of tanycytes in the mediobasal hypothalamus: these modified glial cells line the walls of the 3rd ventricle, and serve a gating function modulating the influence of nutrients, hormones and metabolites as feedback signals on neuronal populations involved in appetite, energy balance and reproduction (Balland et al., 2014; Bolborea et al., 2020; Frayling et al., 2011; Langlet, 2014; Langlet et al., 2013; Lhomme et al., 2021). Tanycytes have been linked to circadian control of glucose homeostasis (Rodríguez-Cortés et al., 2022) and have emerged as a key cellular substrate for seasonal modulation of hypothalamic function (Dardente et al., 2014, 2019; Hazlerigg and Loudon, 2008). This latter aspect depends on photoperiod-dependent production of thyrotropin by neighbouring pars tuberalis cells, which in turn acts via tanycytic thyroid-stimulating hormone (TSH) receptor expression (Hanon et al., 2008). This then modulates tanycytic uptake of thyroid hormone by monocarboxylate transporter 8 (SLC16A2) (Friesema et al., 2003; Petri et al., 2016) and tanycytic conversion of thyroid hormone by deiodinases (dio2/dio3) (Dardente et al., 2014, 2019; Hazlerigg and Loudon, 2008). The relevance of this pathway for expression of torpor is suggested by the blockade of daily torpor expression in Siberian hamsters by exogenous thyroid hormone delivery to the dorsomedial hypothalamus (Murphy et al., 2012), a site to which tanycyte cell processes project (Fig. 3H) (Guerra et al., 2010; Güldner and Wolff, 1973; Rodríguez et al., 2005). Furthermore, in the Arctic ground squirrel, circannual termination of hibernation correlates with increased deiodinase 2 gene expression in the mediobasal hypothalamus (Chmura et al., 2022).

Hence, it can be seen that involvement in temporal changes in metabolic energy demand and thyroid metabolism constitutes a unifying functional framework for the three non-neuronal tissues in which we see peak *c-fos* expression at arousal initiation. This leads us to hypothesise that *c-fos* induction occurs in response to metabolic changes developing during torpor, reflecting the direct sensitivity of these tissues to changing concentrations of blood- or CSF-borne metabolites. Further experiments are required to test this hypothesis and to explore how changes in tanycyte or choroid plexus activity might couple to the neural circuits driving thermogenesis at arousal.

## Supplementary Material

10.1242/jexbio.247224_sup1 Supplementary information
