# Supplementary material for: c-fos induction in the choroid plexus, tanycytes and pars tuberalis is an early indicator of spontaneous arousal from torpor in a deep hibernator
Source: J Exp Biol. 2024 May 23;227(10):jeb247224. doi: 10.1242/jeb.247224 (PMC11166454; doi:10.1242/jeb.247224)
Supplement: Supplementary information [file jexbio-227-247224-s1.pdf]

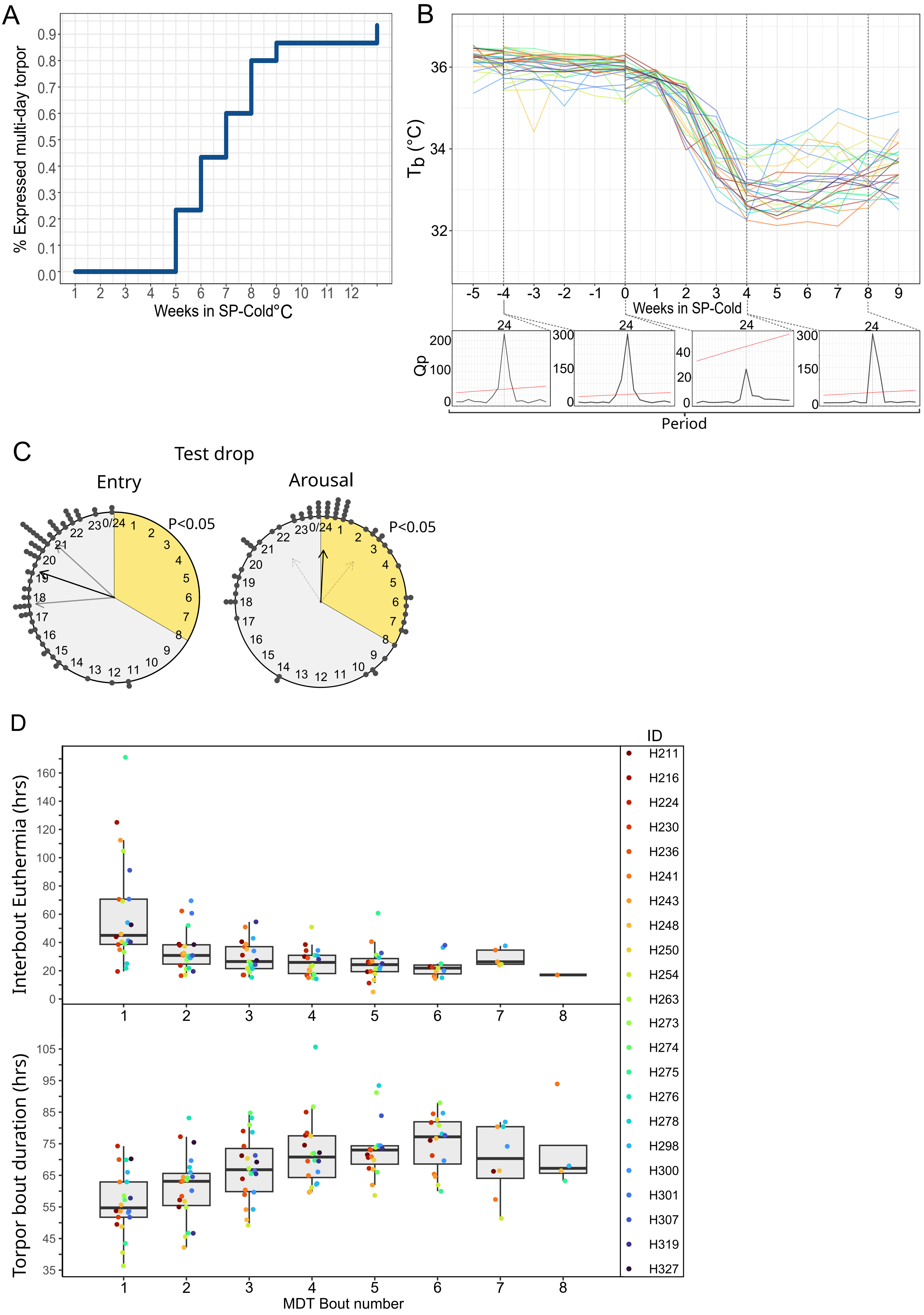

Journal of Experimental Biology • Supplementary information

Fig. S1. Physiological monitoring of hibernation in the golden hamster

A. Percentage of animals hibernating in response to SP-cold conditions over time.

B. Core body temperature (T_b) in response to the transition from long photoperiod (LP) at ambient temperature (T_a) 21°C, to short photoperiod (SP) at T_a 21°C (first dotted horizontal line), to SP and T_a 7.4°C. Prior to T-A cycling diel patterning of mean core body temperature was observed at all stages of the experiment, except the first 4 weeks of cold where the T_b is reduced (see inset periodograms). Periodograms were generated using ActogramJ and the chi-square method.

C. Raleigh plots showing the time of test-drop entry and arousal from 24 individuals. Photoperiod is represented by grey for dark and yellow for light. The black dots are an individual arousal or entry event. The black arrow indicates the mean time of entry or arousal, and the light grey arrows indicates the standard deviation. The length of the arrow represents mean resultant length as an indicator of concentration around the mean, therefore reflecting the statistical significance. The Raleigh statistical was used to test for a time-of-day preference in arousal and entry. Both entry and arousal were significant $p<0.05$.

D. Top: Duration (hours) of inter bout euthermia after previous corresponding torpor bout for 22 individuals; listed on the side with individual colours shown in each box plot. Bottom: Torpor bout duration (hours) for each torpor bout made.

Journal of Experimental Biology • Supplementary information

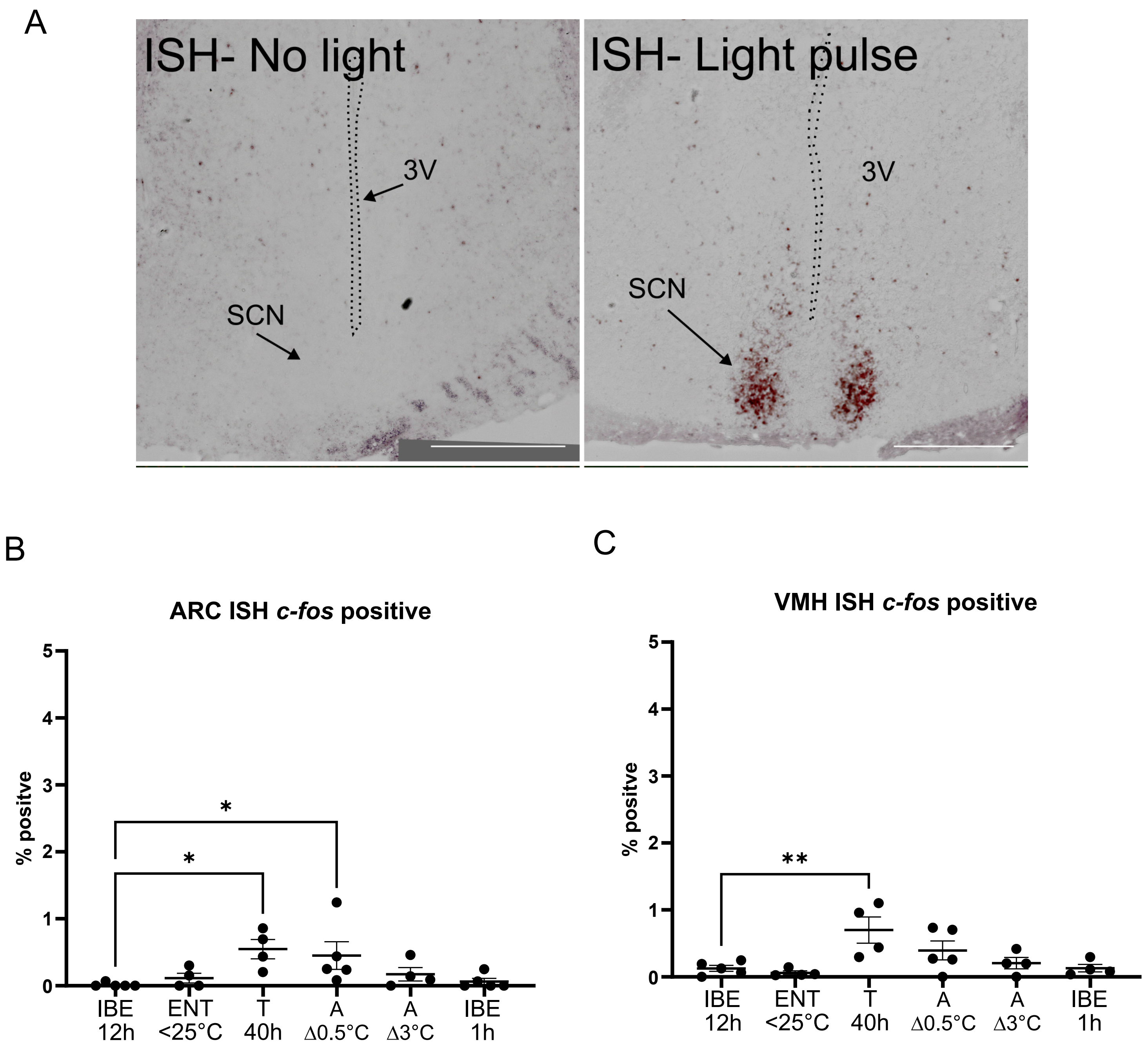

Fig. S2. Validation of *c-fos* probe and numbers of *c-fos* positive cells in the ARC and VMH

A. In-situ hybridization of the suprachiasmatic nucleus (SCN) in the golden hamster for *c-fos* RNA (brown staining). Left: no light pulse during the dark phase. Right: 20-minute light pulse during the dark phase. 3V; 3rd ventricle, indicated by black dotted line.

B. Quantification of the percentage of positive cells in the arcuate nucleus (ARC) region. Each dot represents one animal, with 3 sections per animal quantified. Error bars represent the standard error of the mean (SEM). Results of one-way ANOVA and post hoc testing by Dunnetts multiple comparisons test are shown; * p-value ≤0.05.

C. Quantification of the percentage of positive cells in the ventromedial hypothalamus (VMH) region. Each dot represents one animal, with 3 sections per animal quantified. Error bars represent the standard error of the mean (SEM). Results of one-way ANOVA and post hoc testing by Dunnetts multiple comparisons test are shown; ** p - value ≤0.005.

Journal of Experimental Biology • Supplementary information

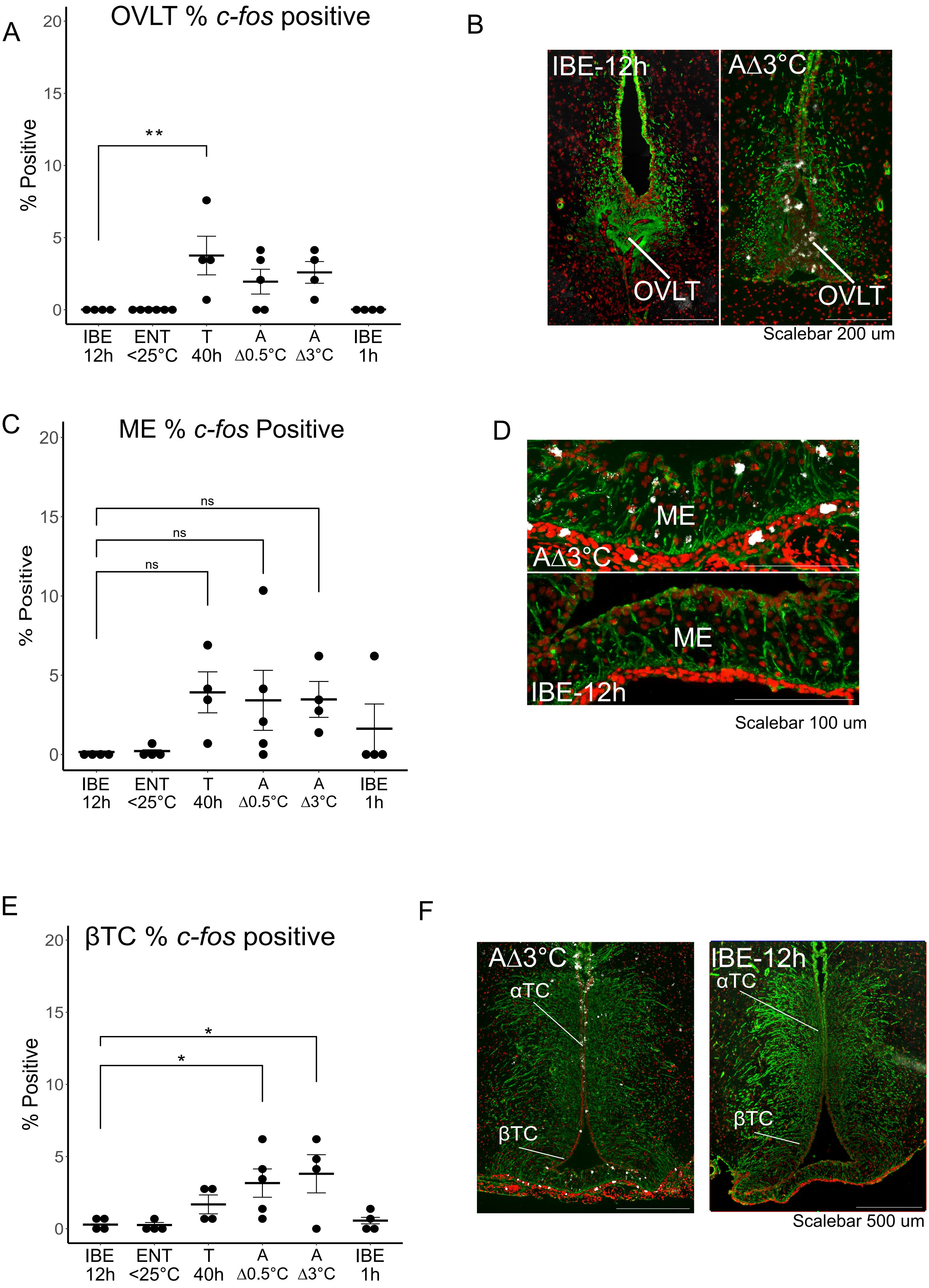
A
OVLT % c-fos positive
% Positive
**
IBE 12h
ENT <25°C
T 40h
A Δ0.5°C
A Δ3°C
IBE 1h
B
IBE-12h
AΔ3°C
OVLT
Scalebar 200 um
C
ME % c-fos Positive
ns
D
ME
Scalebar 100 um
E
βTC % c-fos positive
*
F
αTC
βTC
Scalebar 500 um

Fig. S3. *C-fos* expression in the OVLT, ME and beta tanycytes

A. Quantification of the percentage of positive cells in the Vascular organ of lamina terminalis (OVLT) region. Each dot represents one animal, with 3 sections per animal quantified. Error bars represent the standard error of the mean (SEM). Results of one-way ANOVA and post hoc testing by Dunnetts multiple comparisons test are shown; ** p-value ≤0.005.

B. Representative images of in-situ hybridization using *c-fos* (white) and sytox orange to show nuclei (red) in the OVLT region, for IBE-12h and AΔ3°C. Vimentin staining is shown in green. Scalebar 200 µm.

C. Quantification of the percentage of positive cells in the median eminence (ME). Each dot represents one animal, with 3 sections per animal quantified. Error bars represent the standard error of the mean (SEM). Results of one-way ANOVA and post hoc testing by Dunnetts multiple comparisons test are shown; ns = not significant.

D. Representative images of in-situ hybridization using *c-fos* (white) and sytox orange to show nuclei (red) in the ME region, for IBE-12h and AΔ3°C. Vimentin staining is shown in green. Scalebar 100 µm.

E. Quantification of the percentage of positive cells in the β-tanycytes (βTC) region. Each dot represents one animal, with 3 sections per animal quantified. Error bars represent the standard error of the mean (SEM). Results of one-way ANOVA and post hoc testing by Dunnetts multiple comparisons test are shown; * p-value ≤0.05.

F. Representative images of in-situ hybridization using *c-fos* (white) and sytox orange to show nuclei (red) in the β-tanycytes (βTC) region, for IBE-12h and AΔ3°C. Vimentin staining is shown in green. Scalebar 500 µm.
